# Supplementary material for: Resilient anatomy and local plasticity of naive and stress haematopoiesis
Source: Nature. 2024 Mar 20;627(8005):839–46. doi: 10.1038/s41586-024-07186-6 (PMC10972750; doi:10.1038/s41586-024-07186-6)
Supplement: Supplementary file 1 — This file contains Supplementary Figs. 1–11. [file 41586_2024_7186_MOESM1_ESM.pdf]

nature portfolio

https://doi.org/10.1038/s41586-024-07186-6

Supplementary information

Resilient anatomy and local plasticity of naive and stress haematopoiesis

In the format provided by the authors and unedited

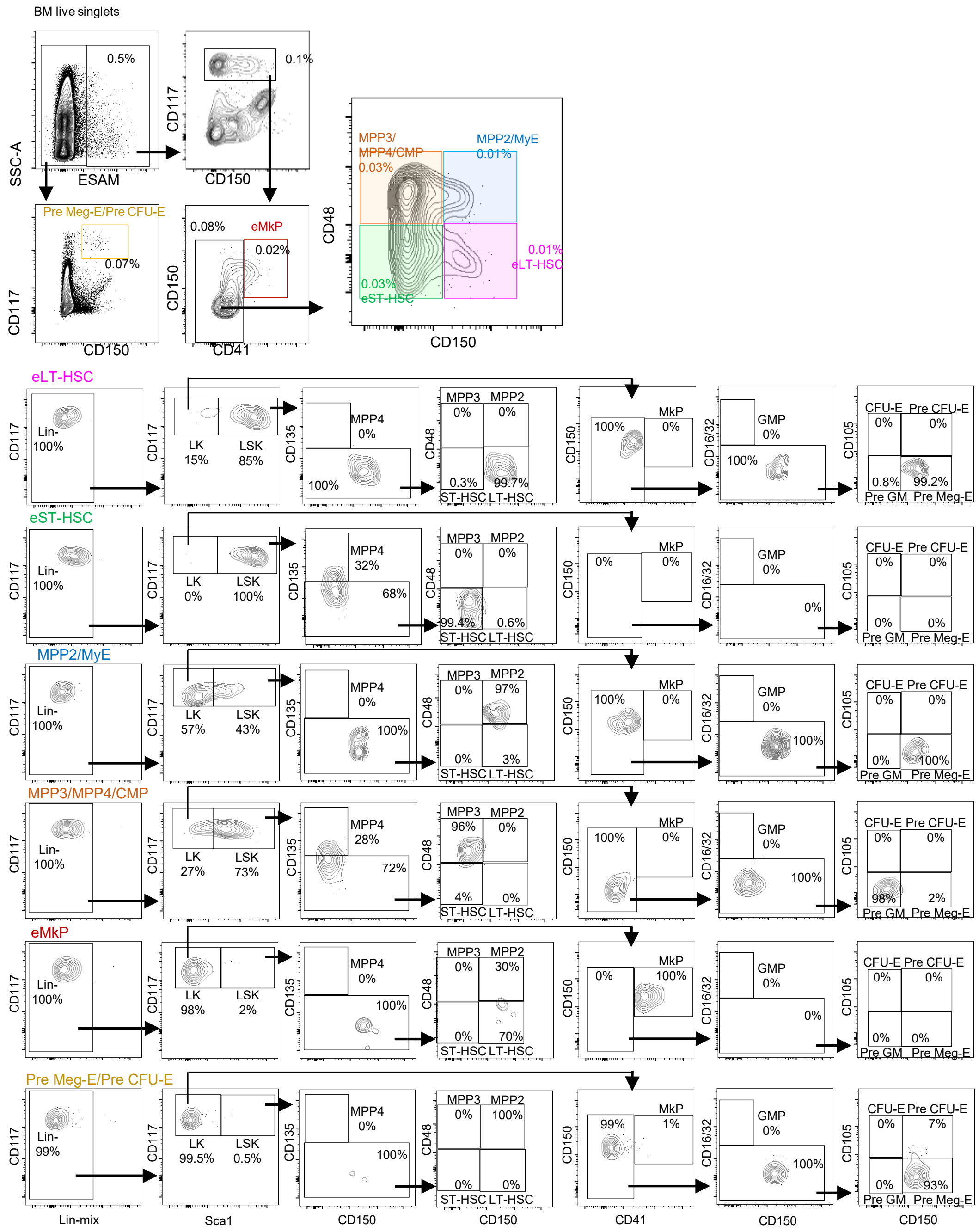

Supplementary Figure 1. FACS plots showing the relative purity of ESAM⁺ HSPC. FACS plots showing the gating strategy to identify 14 indicated hematopoietic progenitors (LT-HSC, ST-HSC, MPP2, MPP3 and MPP4 are identified as described by Pietras et al.[18]; MkP, Pre Meg-E, Pre CFU-E, CFU-E and Pre GM are identified as described by Pronk et al.[16]; GMP, GP, MDP and MP+cMoP are identified as described by Yanez, A. et al.[17]) that were used for immunophenotyping screen in Fig.1.

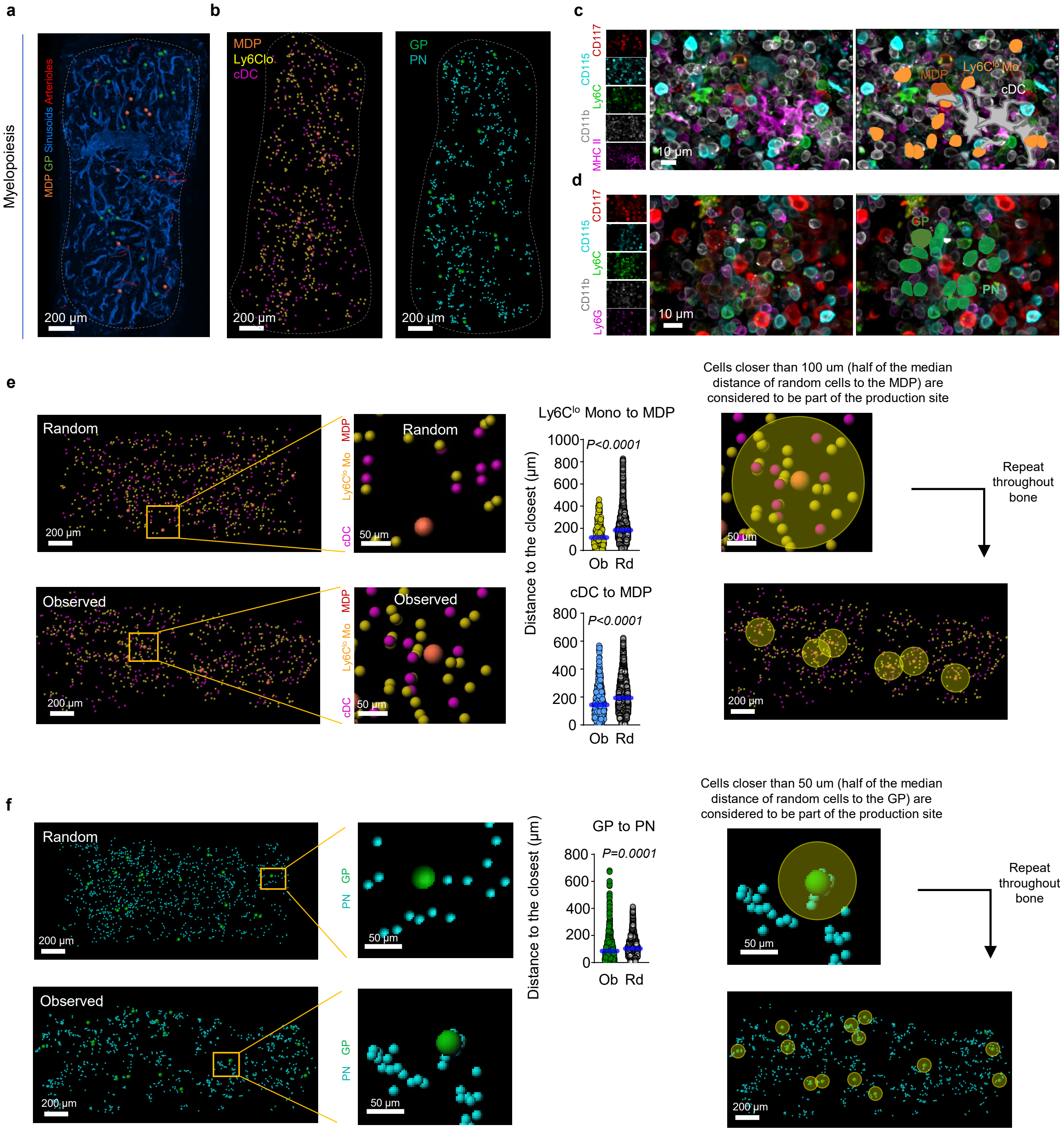

Supplementary Figure 2. Mapping granulopoiesis and Mono/DC-poiesis in whole mounted sternum segments. a, Mapping GP and MDP with vessels. **b,** Mapping Mono/DC-poiesis and granulopoiesis in whole mounted sternum segments. **c,d,** High-power image showing the distribution of Ly6C^{lo} Mono and cDC around MDP (c) and PN around GP (d). **e,** Identification of Mono/Dendritic production sites. In contrast to random cells MDP cluster together with Ly6C^{lo} Monocytes and dendritic cells. The mean distance for random cells to the closest MDP was 203 µm for Ly6C^{lo} monocytes, and 207 µm for cDC. Based on this we defined each production site as Ly6Clo monocytes and cDC cluster tightly, cells closer than 100 µm (half of the median distance of random cells to the MDP) to be part of the production site; These analyses are then repeated through the bone to identify all production sites in the section. **f,** Identification of Granulopoiesis production sites. In contrast to random cells GP cluster together with PN cells. The mean distance for random cells to the closest GP was 113 µm for PN cells. Based on this we defined each production site as cells closer than 50 µm (half of the median distance of random cells to the GP) to be part of the production site; These analyses are then repeated through the bone to identify all production sites in the section. In the rare cases when the edges of two production sites overlap the cells were assigned to the closest of the two production sites. After stress (infection, G-CSF, or aging) we found numerous GP that were close to each other (<20µm). In this case we considered them to be part of the same production site. At least three times each experiment was repeated independently with similar results. Statistical differences were calculated using two-tailed unpaired Student's *t*-tests; *P* values are shown.

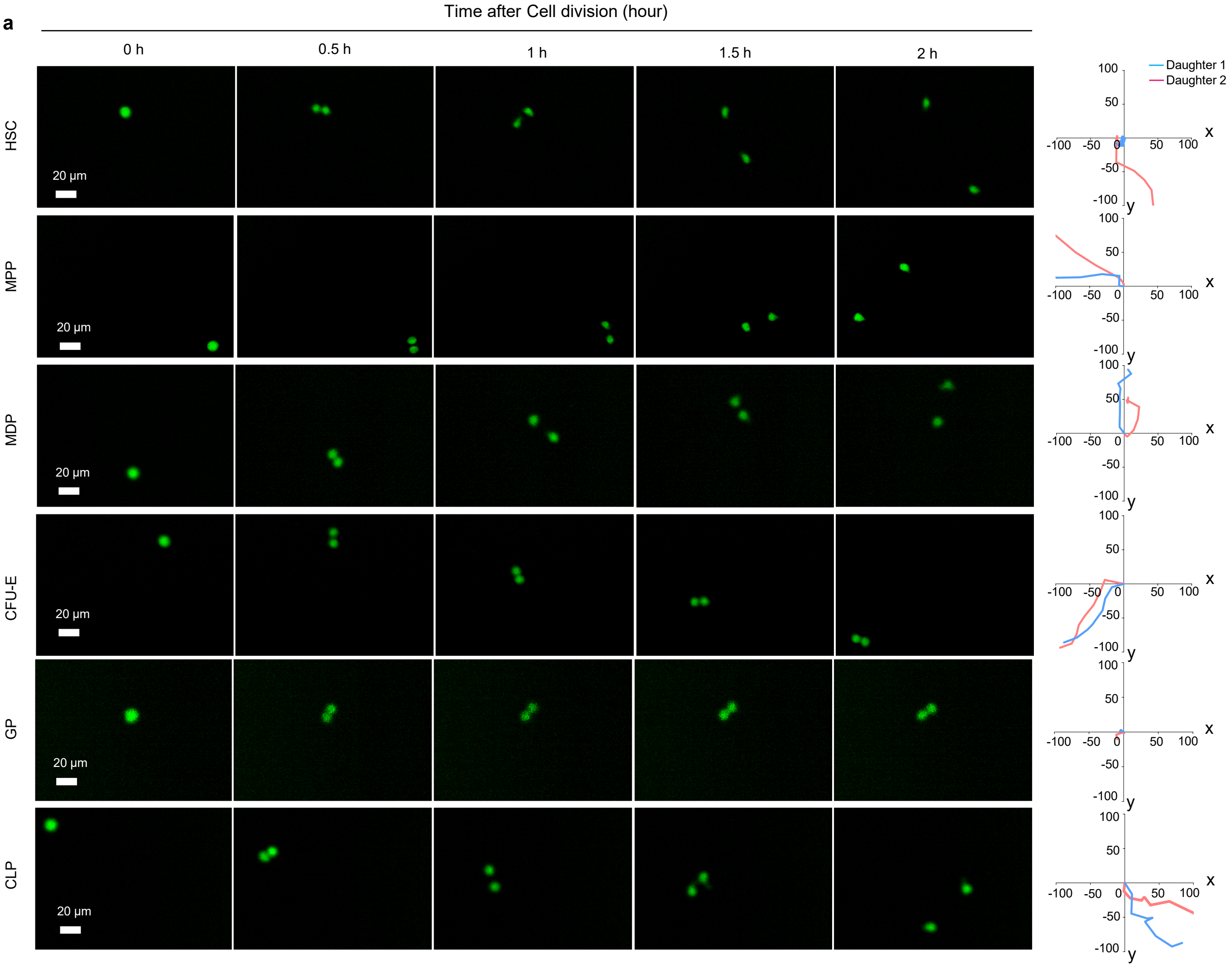

Supplementary Figure 3. Live imaging analyses of hematopoietic behavior after cell division. a, the microphotograph sequences show the localization of a single GFP^{+} stem or progenitor cell of interest (t=0, just before cell division) and their offspring (t=0.5-2 hours after cell division). The right panels show the individual trajectory of each daughter cell (red or blue lines) over a period of two hours after cell division (x=0, y=0 are the coordinates, in microns, of the mother cell at fission). n =30 HSC, MPP, CFU-E, or MDP and n=18 GP in 5 independent experiments.

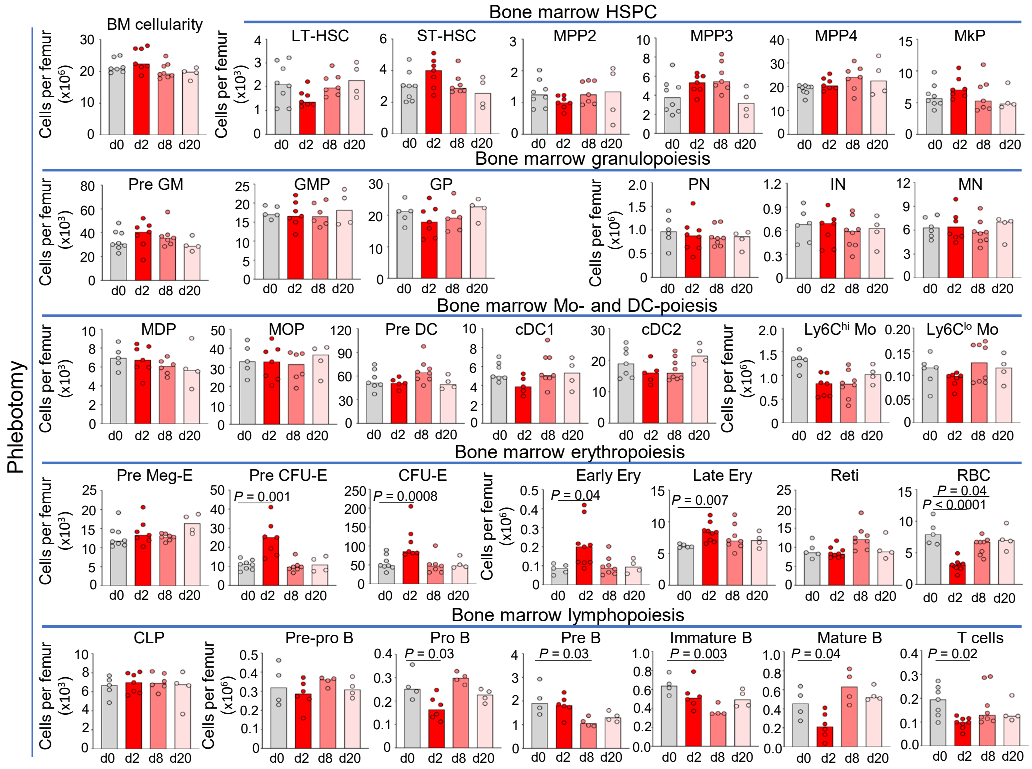

Supplementary Figure 4. FACS analyses of the hematopoietic response to phlebotomy. Number of the indicated cells per femur (detected by FACS. Bone marrow HSPC populations are identified as in Extended Data Figure 1a; granulopoiesis and Mo- and DC-poiesis populations are identified as described in reference[1]; erythropoiesis populations are identified as described in Extended Data Figure 2 d and f; B lymphopoiesis populations are identified as described as in Extended Data Figure 2i) at the indicated time points after phlebotomy. Each dot corresponds to one mouse in at least 3 independent experiments. n=7, 7, 8 and 4 for total BM cellularity at d0, d2, d8 and d20 per femur after phlebotomy; n=8, 7, 7 and 4 for LT-HSC, ST-HSC, MPP2, MPP3, MPP4, MKP, Pre GM, Pre Meg-E, Pre CFU-E and CFU-E cell numbers per femur; n=5, 9, 8 and 4 for Early Ery, Late Ery, Reticulocytes, RBC cell numbers per femur at d0, d2, d8 and d20 after phlebotomy; n=5, 7, 6, and 4 for CLP, GMP, MDP, MOP, GP cell numbers per femur at d0, d2, d8 and d20 after phlebotomy; n= 6, 7, 8 and 4 for PN, IN, MN, Ly6C^{hi} Mo, Ly6C^{low} Mo cell numbers per femur at d0, d2, d8 and d20 after phlebotomy; n= 7, 5, 8 and 4 for Pre DC, cDC1, cDC2 cell numbers per femur at d0, d2, d8 and d20 after phlebotomy; n=4, 6, 4 and 4 for Pre-pro B, Pro B, Pre B, Immature B, Mature B cell numbers per femur at d0, d2, d8 and d20 after phlebotomy; n= 7, 8, 8 and 4 for T cell numbers per femur at d0, d2, d8 and d20 after phlebotomy). The bar shows the median. Statistical differences were calculated using Ordinary one-way ANOVA, Dunnett's multiple comparisons test; *P* values are shown.

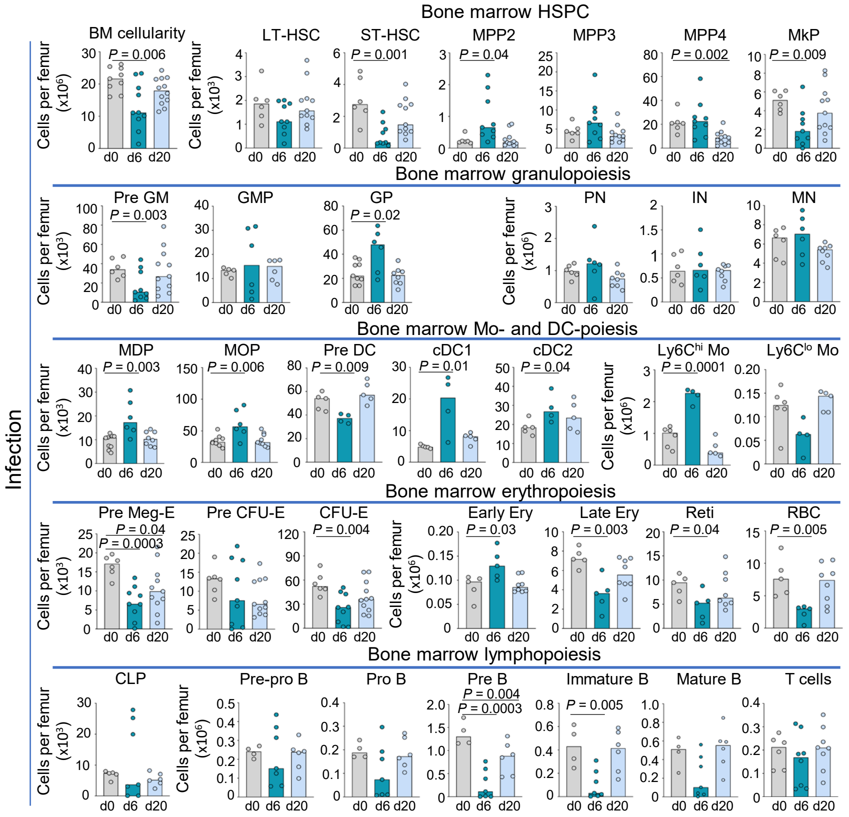

Supplementary Figure 5. FACS analyses of the hematopoietic response to ***L. monocytogenes***. **a,** Number of the indicated cells per femur (detected by FACS. Bone marrow HSPC populations are identified as in Extended Data Figure 1a; granulopoiesis and Mo- and DC-poiesis populations are identified as described in reference[1]; erythropoiesis populations are identified as described in Extended Data Figure 2 d and f; B lymphopoiesis populations are identified as described as in Extended Data Figure 2i) at the indicated time points after *L. monocytogenes* infection. Each dot corresponds to one mouse in at least 3 independent experiments （n=9, 10 and 12 for BM cellularity per femur at d0, d2 and d20 after *L. monocytogenes* infection; n=6, 9 and 11 for LT-HSC, ST-HSC, MPP2, MPP3, MPP4, MkP, Pre GM, Pre Meg-E, Pre CFU-E, CFU-E cell numbers per femur at d0, d2 and d20 after *L. monocytogenes* infection; n=5, 6 and 6 for GMP cell numbers per femur at d0, d2 and d20 after *L. monocytogenes* infection; n=9, 6 and 8 for MDP, MoP, GP cell numbers per femur at d0, d2 and d20 after *L. monocytogenes* infection; n=6, 6 and 8 for PN, IN, MN cell numbers per femur at d0, d2 and d20 after *L. monocytogenes* infection; n= 6, 4 and 5 for $Ly6C^{low}$ and $Ly6C^{hi}$ cell numbers per femur at d0, d2 and d20 after *L. monocytogenes* infection; n=5, 4, and 5 for pre DC, cDC1, cDC2 cell numbers per femur at d0, d2 and d20 after *L. monocytogenes* infection; n=5, 5, and 8 for Early Ery, Late Ery, Reticulocytes, RBC cell numbers per femur at d0, d2 and d20 after *L. monocytogenes* infection; n=4, 7, and 6 for Pre-pro B, Pro B, Pre B, Immature B, Mature B cell numbers per femur at d0, d2 and d20 after *L. monocytogenes* infection; n=6, 8 and 8 for T cell numbers per femur at d0, d2 and d20 after *L. monocytogenes* infection). The bar shows the median. Statistical differences were calculated using Ordinary one-way ANOVA, Dunnett's multiple comparisons test; *P* values are shown.

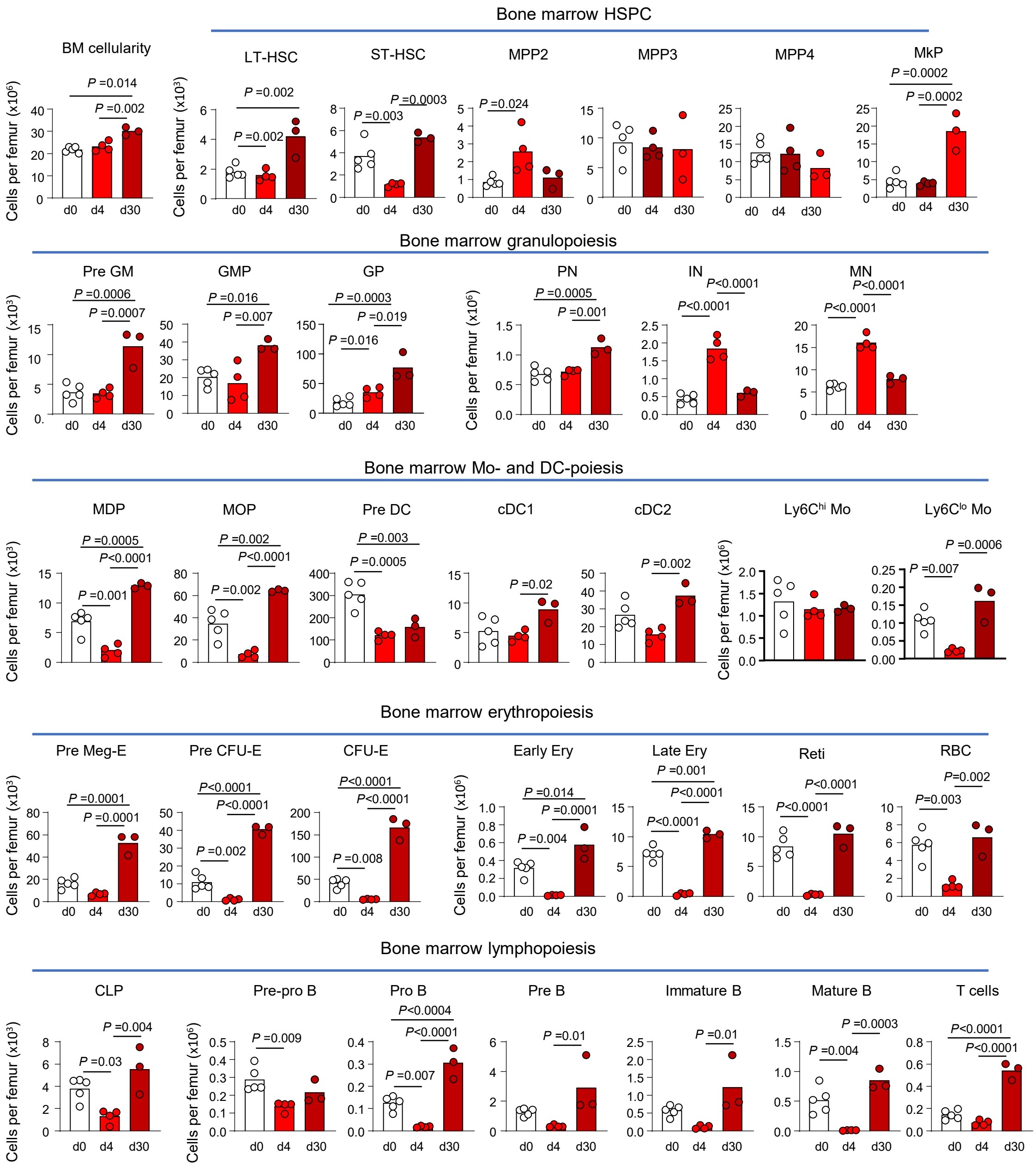

Supplementary Figure 6. FACS analyses of the hematopoietic response to G-CSF. Number of the indicated cells per femur (detected by FACS). Bone marrow HSPC populations are identified as in Extended Data Figure 1a; granulopoiesis and Mo- and DC-poiesis populations are identified as described in reference[1]; erythropoiesis populations are identified as described in Extended Data Figure 1 m and o; B lymphopoiesis populations are identified as described as in Extended Date Figure 1r) at the indicated time points (4 days of G-CSF treatment, 30 days after G-CSF treatment). Each dot corresponds to one mouse in at least 3 independent experiments. (n=5, 4 and 3 for all the indicated cell populations at the indicated time points (d0 for no treatment, d4 for 4 days of G-CSF treatment, day 30 for 30 days after G-CSF treatment). The bar shows the median. Statistical differences were calculated using Ordinary one-way ANOVA, Dunnett's multiple comparisons test; *P* values are shown.

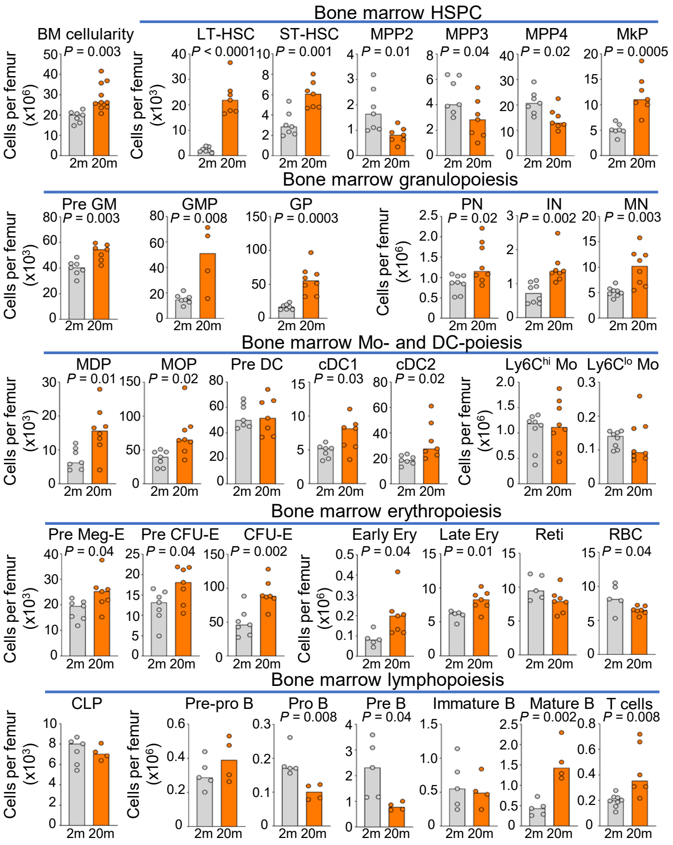

Supplementary Figure 7. FACS analyses comparing hematopoiesis in 2- and 20-month old mice. Number of the indicated cells per femur (detected by FACS. Bone marrow HSPC populations are identified as in Extended Data Figure 1a; granulopoiesis and Mo- and DC-poiesis populations are identified as described in reference[1]; erythropoiesis populations are identified as described in Extended Data Figure 1 m and n; B lymphopoiesis populations are identified as described as in Extended Date Figure 1r) in 2-month-old (n = 7 for BM cellularity, LT-HSC, ST-HSC, MPP2, MPP3, MPP4, MkP, Pre GM, GMP, GP, MDP, MoP, Pre DC, cDC1, cDC2, Pre Meg-E, Pre CFU-E, CFUE cell numbers per femur; n=8 for PN, IN, MN, Ly6C^{hi}, Ly6C^{low}, T cell numbers per femur; n=5 for Early Ery, Late Ery, Reticulocytes, RBC, CLP, Pre-pro B, Pro B, Pre B, Immature B, Mature B cell numbers per femur) and 20-month-old mice (n=10 for BM cellularity per femur; n=7 for LT-HSC, ST-HSC, MPP2, MPP3, MkP, Pre GM, Pre DC, cDC1, cDC2, Pre Meg-E, Pre CFU-E, CFU-E, Early Ery, Late Ery, Reticulocytes, RBC cell numbers per femur; n=8 for GP, PN, IN, MN, MDP, MoP, Ly6C^{hi}, Ly6C^{low} cell numbers per femur; n=4 for GMP, CLP, Pre-pro B, Pro B, Pre B, Immature B, Mature B cell numbers per femur; n=6 for T cell number per femur). Each dot corresponds to one mouse in 3 independent experiments. All data represent individual values with median plot. Statistical differences were calculated using two-tailed unpaired Student's *t*-tests; *P* values are shown.

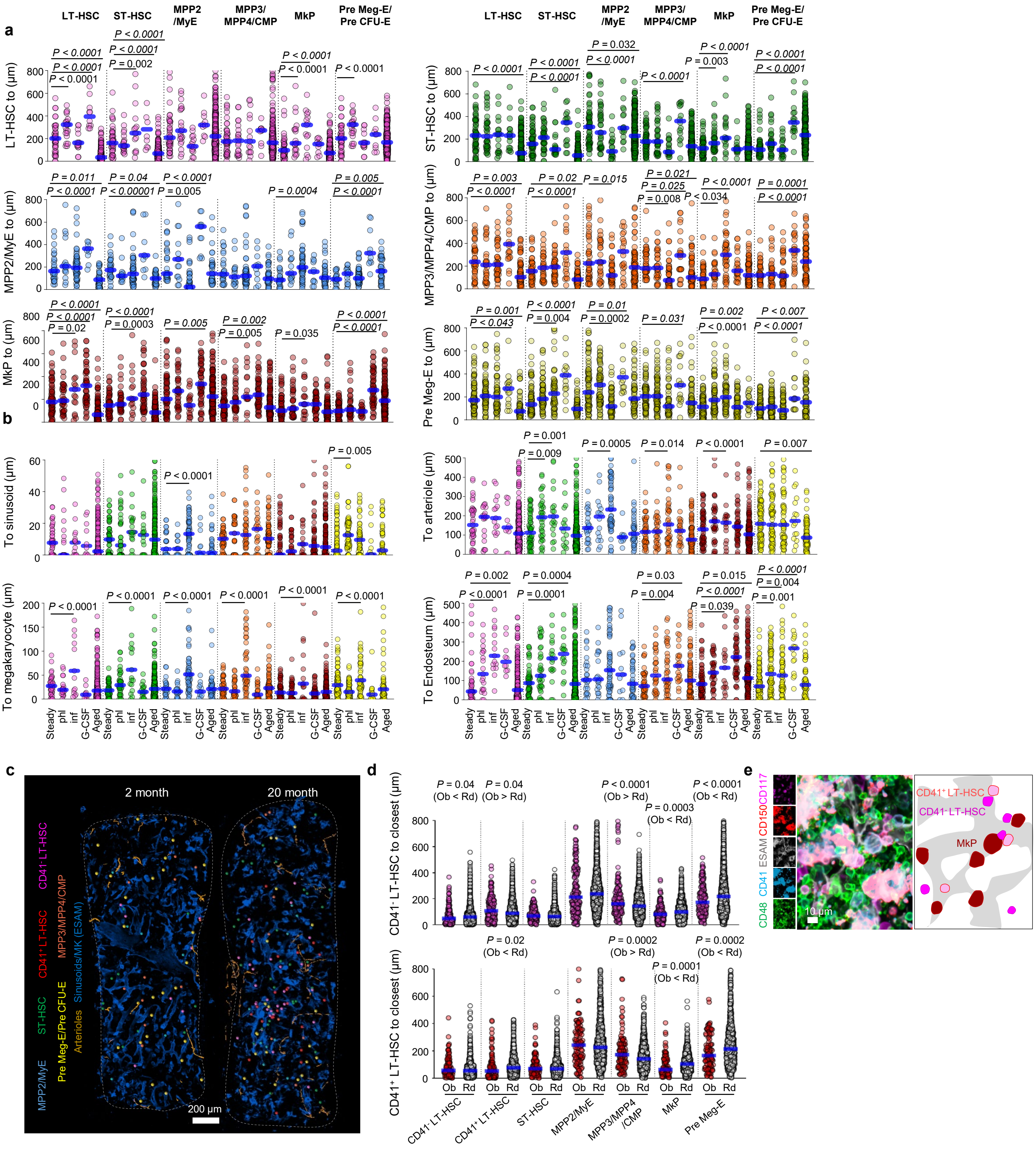

Supplementary Figure 8. Myeloid and Lymphoid biased LT-HSC distribution in aged mice. a,b, Distance from each HSPC to all other indicated cells (a) and structures (b) at at the indicated time points after insult. (n = 41 LT-HSC, 52 ST-HSC, 25 MPP2/MyE, 41 MPP3/MPP4/CMP, 61 MkP, and 82 Pre Meg-E in 4 sternum segments from four mice in steady-state; n = 21 LT-HSC, 35 ST-HSC, 16 MPP2/MyE, 30 MPP3/MPP4/CMP, 38 MkP, and 73 Pre Meg-E in 3 sternum segments from three mice two days after phlebotomy; n = 15 LT-HSC, 19 ST-HSC, 56 MPP2/MyE, 39 MPP3/MPP4/CMP, 17 MkP, and 57 Pre Meg-E in 3 sternum segments from three mice six days after infection; n = 10 LT-HSC, 16 ST-HSC, 11 MPP2/MyE, 23 MPP3/MPP4/CMP, 52 MkP, and 16 Pre Meg-E in 3 sternum segments from three mice four days of G-CSF treatment; n = 300 LT-HSC, 236 ST-HSC, 39 MPP2/MyE, 72 MPP3/MPP4/CMP, 133 MkP, and 57 Pre Meg-E in 3 sternum segments from three 20-month old mice.LT-HSC in aged mice contain CD41+ and CD41- LT-HSC identified as shown in the next panels. Statistical differences were calculated using two way ANOVA t-tests if the distributions were normal or Kruskal-Wallis test if not normal; P values are shown. **c,** Maps showing changes in HSPC distribution with age. Map dots are three times the average size of the relevant cell. **d**, Distances analyses from each $CD41^-$ or $CD41^+$ (n = 191 or 109 in 3 sternum segments from 3 mice) LT-HSC to all other HSPC. Statistical differences were calculated using two-tailed unpaired Student's t-tests; *P* values are shown. **e,** Representative images showing an LT-HSC cluster in a 20-month-old sternum.

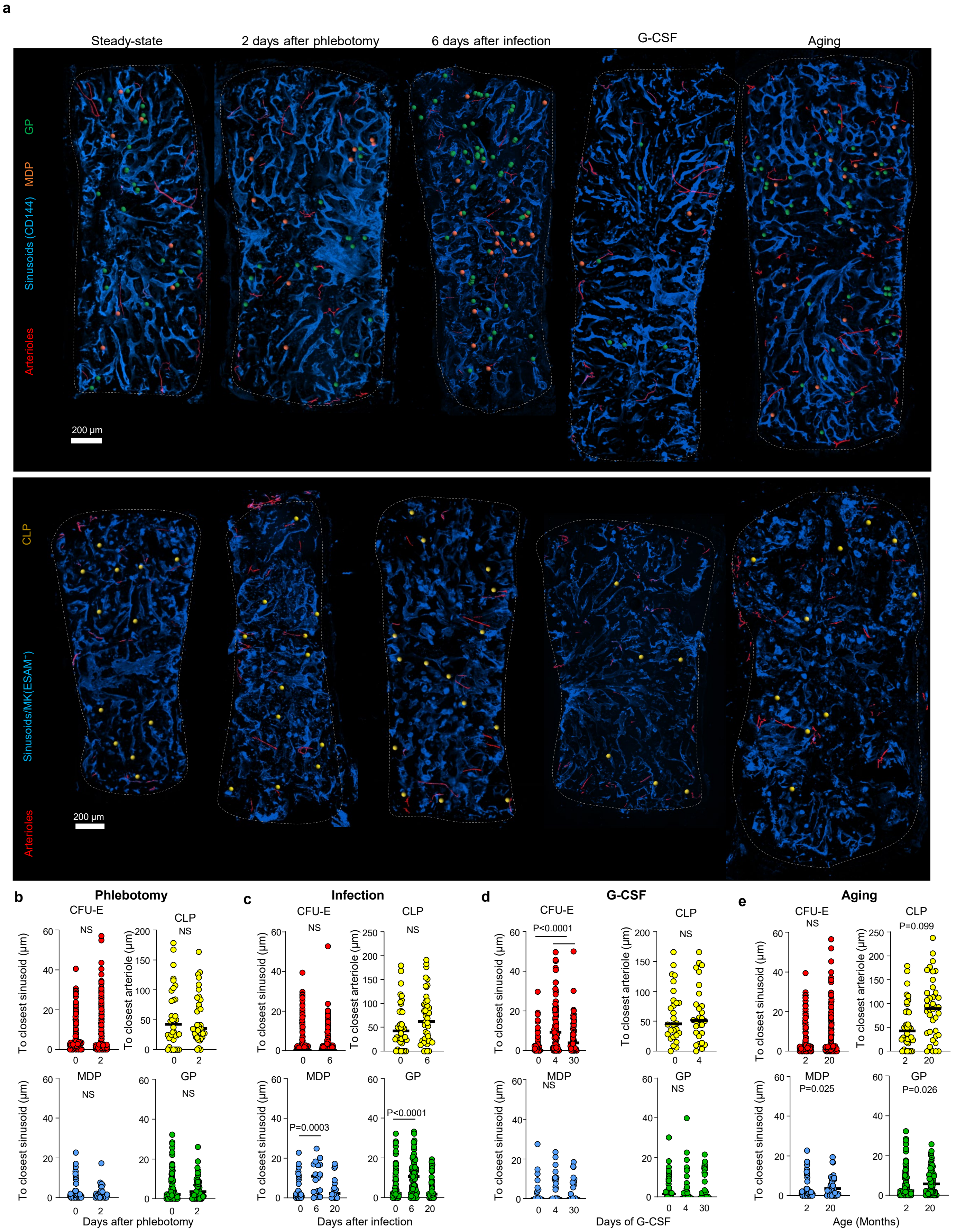

Supplementary Figure 9. Lineage-committed progenitors location and distance to arterioles or sinusoids. See full legend on next page

Supplementary Figure 9. Lineage-committed progenitors location and distance to arterioles or sinusoids. **a,** Maps showing changes in lineage-committed progenitors distribution in steady-state, two days after phlebotomy, six days after infection, four days of G-CSF treatment and aging. Map dots of CLP are five times and other dots are three times the average size of the relevant cell. **b,** Distance analyses from each indicated progenitor to the closest indicated vessel at the indicated time points. (n = 315 CFU-E, 62 MDP, 114 GP and 36 CLP in 3 sternum segments from 3 mice in steady-states; n = 870 CFU-E, 25 MDP, 56 GP and 34 CLP in 3 sternum segments from 3 mice 2 days after phlebotomy; n = 314 CFU-E, 16 MDP, 98 GP and 43 CLP in 3 sternum segments from 3 mice 6 days after infection; n = 18 MDP and 49 GP in 3 sternum segments from 3 mice 20 days after infection; n = 90 CFU-E, 21 MDP, 51 GP and 28 CLP in 3 sternum segments from 3 mice after 4 days of saline treatment as G-CSF control; n = 90 CFU-E, 33 MDP, 46 GP and 28 CLP in 3 sternum segments from 3 mice after 4 days of G-CSF treatment; n = 90 CFU-E, 24 MDP and 43 GP in 3 sternum segments from 3 mice after 30 days of G-CSF treatment).

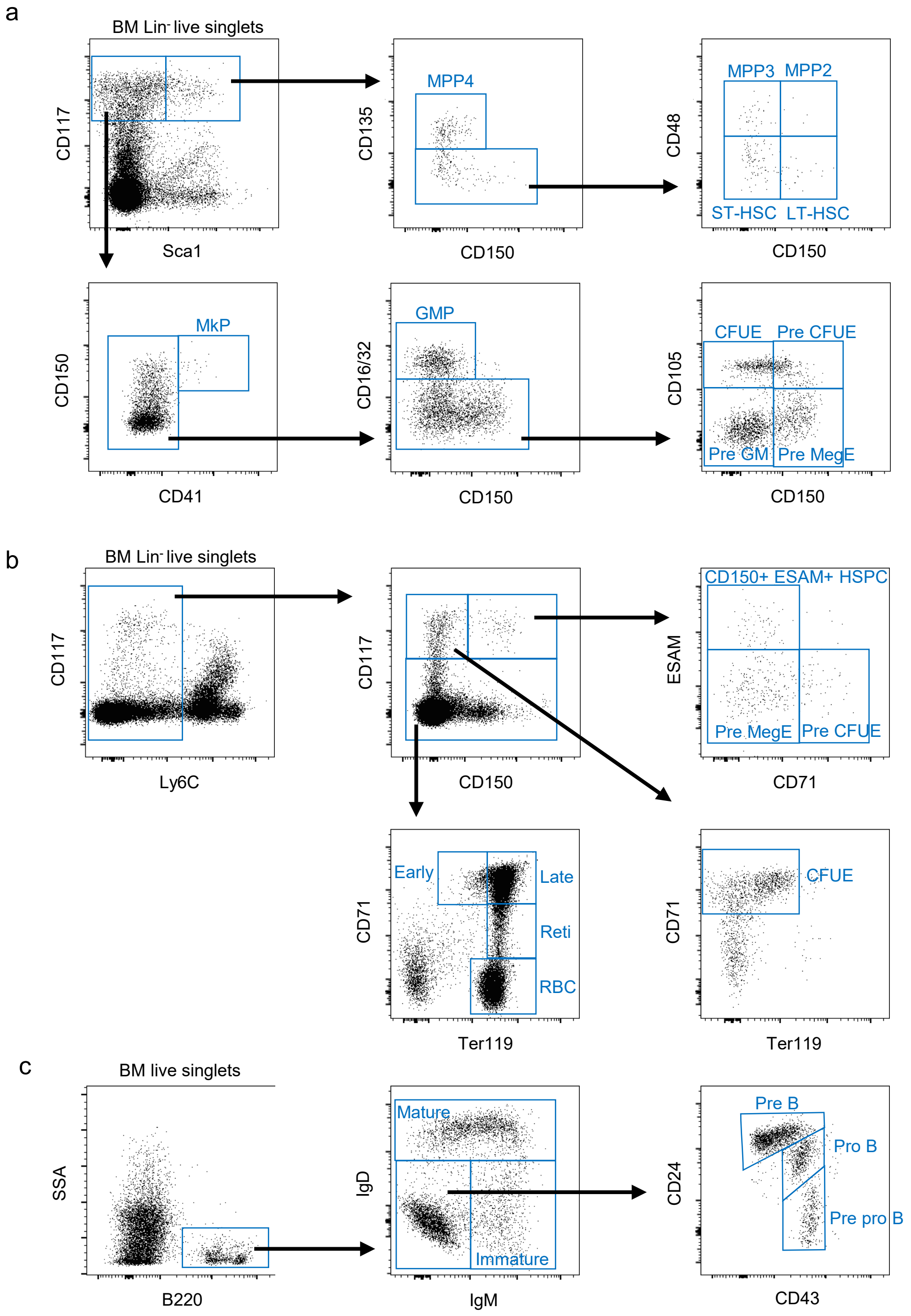

Supplementary Figure 10. FACS gating strategy for isolation of the indicated cells. a, b, c FACS plots showing the gating strategies used for (a) HSPC and Erythroid progenitors –as previously described [16,18], (b) for erythropoiesis with ESAM and previously described [22] and (c) for lymphopoiesis as previously described [23,] - to interrogate the indicated progenitors cell numbers analyzed by flow cytometry assay in Figure 4-5, and Supplementary Figure 4-7.

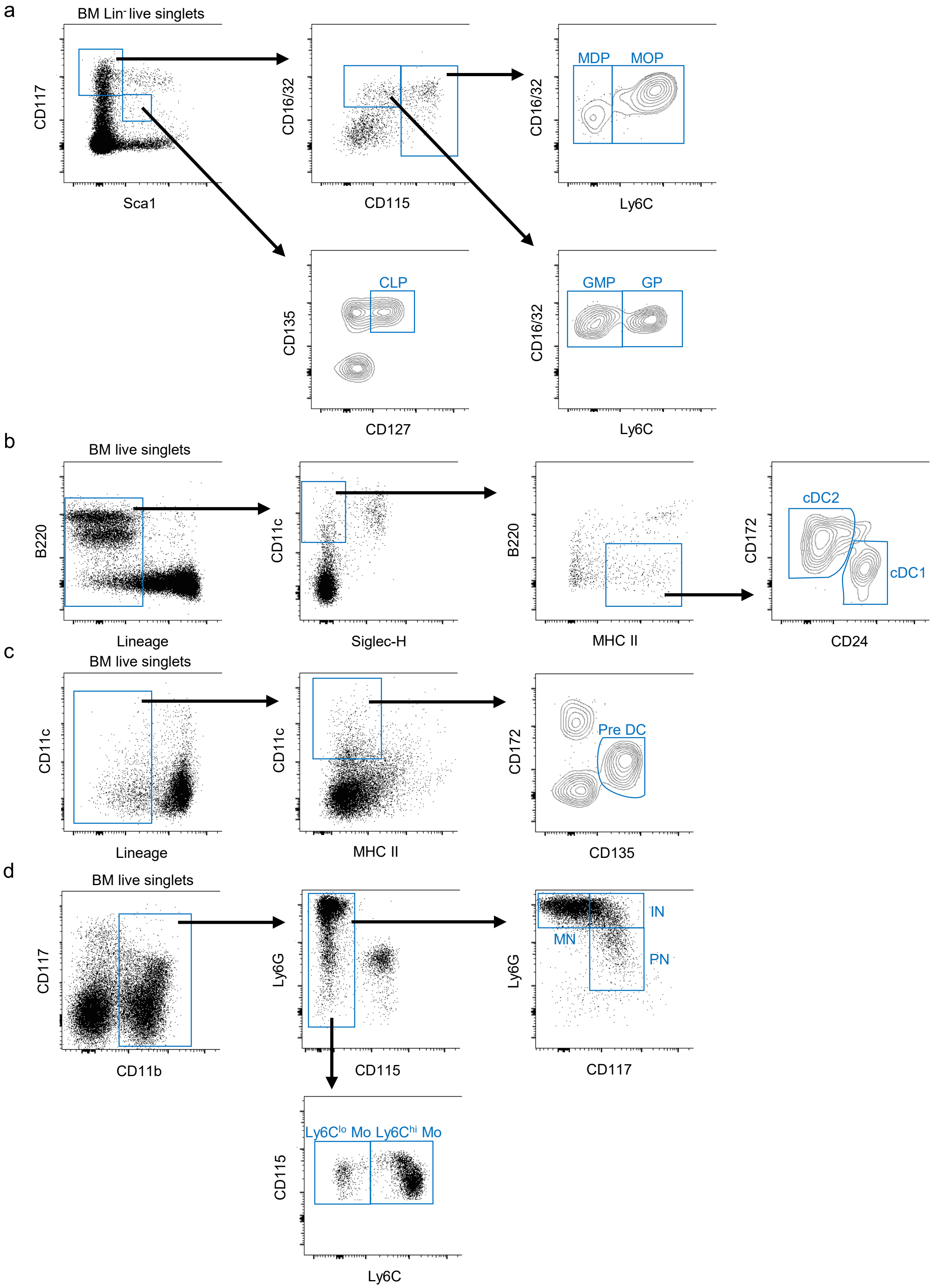

Supplementary Figure11. FACS gating strategy for isolation of the indicated cells. a, b, c FACS plots showing the gating strategies used indicated cells as previously described [1,8]- to interrogate the indicated progenitors cell numbers analyzed by flow cytometry assay in Figure 4-5, and Supplementary Figure 4-7.
