# Supplementary material for: Resilient anatomy and local plasticity of naive and stress haematopoiesis
Source: Nature. 2024 Mar 20;627(8005):839–46. doi: 10.1038/s41586-024-07186-6 (PMC10972750; doi:10.1038/s41586-024-07186-6)
Supplement: Supplementary file 2 — Reporting Summary [file 41586_2024_7186_MOESM2_ESM.pdf]

nature portfolio

Corresponding author(s): Qingqing Wu, Daniel Lucas

Last updated by author(s): 12/01/2023

Reporting Summary

Nature Portfolio wishes to improve the reproducibility of the work that we publish. This form provides structure for consistency and transparency in reporting. For further information on Nature Portfolio policies, see our Editorial Policies and the Editorial Policy Checklist.

Statistics

For all statistical analyses, confirm that the following items are present in the figure legend, table legend, main text, or Methods section.

n/a	Confirmed	
☐	☒	The exact sample size (*n*) for each experimental group/condition, given as a discrete number and unit of measurement
☐	☒	A statement on whether measurements were taken from distinct samples or whether the same sample was measured repeatedly
☐	☒	The statistical test(s) used AND whether they are one- or two-sided<br>*Only common tests should be described solely by name; describe more complex techniques in the Methods section.*
☒	☐	A description of all covariates tested
☒	☐	A description of any assumptions or corrections, such as tests of normality and adjustment for multiple comparisons
☐	☒	A full description of the statistical parameters including central tendency (e.g. means) or other basic estimates (e.g. regression coefficient) AND variation (e.g. standard deviation) or associated estimates of uncertainty (e.g. confidence intervals)
☐	☒	For null hypothesis testing, the test statistic (e.g. *F*, *t*, *r*) with confidence intervals, effect sizes, degrees of freedom and *P* value noted<br>*Give P values as exact values whenever suitable.*
☒	☐	For Bayesian analysis, information on the choice of priors and Markov chain Monte Carlo settings
☒	☐	For hierarchical and complex designs, identification of the appropriate level for tests and full reporting of outcomes
☒	☐	Estimates of effect sizes (e.g. Cohen's *d*, Pearson's *r*), indicating how they were calculated

Our web collection on statistics for biologists contains articles on many of the points above.

Software and code

Policy information about availability of computer code

Data collection	NIS-Elements software (Nikon, version 5.20.02 and 5.30.03). BD FACSDiva software (BD, version 9.0).
Data analysis	NIS-Elements software (Nikon, version 5.20.02 and 5.30.03), Imaris software (Bitplane, version 9.5 to 9.9), and Matlab software (MathWorks, version 2018a) GraphPad Prism software (Graphpad, version 9). FlowJo (Tree Star, version 10).

For manuscripts utilizing custom algorithms or software that are central to the research but not yet described in published literature, software must be made available to editors and reviewers. We strongly encourage code deposition in a community repository (e.g. GitHub). See the Nature Portfolio guidelines for submitting code & software for further information.

Data

Policy information about availability of data

All manuscripts must include a data availability statement. This statement should provide the following information, where applicable:

- Accession codes, unique identifiers, or web links for publicly available datasets
- A description of any restrictions on data availability
- For clinical datasets or third party data, please ensure that the statement adheres to our policy

Source Data for quantifications described in the text or shown in graphs plotted in Figures 1-5 and Extended Data Figures 1-10 are available with the manuscript.
Datasets for all flow analysis and images shown in Figures 1-5 and Extended Data Figures 1-10 are publicly available: "raw image data for "Resilient anatomy and local microplasticity of naïve and stress hematopoiesis "", Mendeley Data, V2, doi: 10.17632/27wvzpyf5h.2

Human research participants

Policy information about studies involving human research participants and Sex and Gender in Research.

Reporting on sex and gender	N/A
Population characteristics	N/A
Recruitment	N/A
Ethics oversight	N/A

Note that full information on the approval of the study protocol must also be provided in the manuscript.

Field-specific reporting

Please select the one below that is the best fit for your research. If you are not sure, read the appropriate sections before making your selection.

☒ Life sciences ☐ Behavioural & social sciences ☐ Ecological, evolutionary & environmental sciences

For a reference copy of the document with all sections, see nature.com/documents/nr-reporting-summary-flat.pdf

Life sciences study design

All studies must disclose on these points even when the disclosure is negative.

Sample size	Because analyses of large bone marrow samples is a very time consuming process (1-6 hours per sample) it is not possible to examine large numbers of samples. We have previously shown that 3 bones per condition allow identification of sufficient numbers of cells to detect changes in location and distribution in the bone marrow (doi:10.1038/s41586-021-03201-2 (2021)). Based on this we have strived to analyze 3 bones per condition. At the request of the referees, we have increased the number of samples for some experiments (e.g. Figure 5).
Data exclusions	all mice were included in the analyses. no data were excluded from the analyses
Replication	Results are representative of at least three independent experiments. All attempts at replication were successful.
Randomization	Mice were randomly allocated to the different groups based on the cage, genotype, and litter size.
Blinding	In this manuscript we have imaged bones from WT (or reporter mice) subjected to different insults and stained with different antibodies. It was not possible to blind the investigator to the type of bone examined as these are readily identified by shape. Similarly, the insults used (hemorrhage, G-CSF, L. monocytogenes infection, and aging) generate such evident changes in cellular content in the bone marrow (hemorrhage, G-CSF, L. monocytogenes infection) or shape of the bone (aged mice, bones become larger) that it was not possible to blind the investigator to the type of insult examined.

Reporting for specific materials, systems and methods

We require information from authors about some types of materials, experimental systems and methods used in many studies. Here, indicate whether each material, system or method listed is relevant to your study. If you are not sure if a list item applies to your research, read the appropriate section before selecting a response.

Materials & experimental systems

n/a	Involved in the study	
☐	☒	Antibodies
☒	☐	Eukaryotic cell lines
☒	☐	Palaeontology and archaeology
☐	☒	Animals and other organisms
☒	☐	Clinical data
☒	☐	Dual use research of concern

Methods

n/a	Involved in the study	
☒	☐	ChIP-seq
☐	☒	Flow cytometry
☒	☐	MRI-based neuroimaging

Antibodies

Antibodies used	B220-APCCy7, BioLegend, catalog number 103224; B220-AF488, BioLegend, catalog number 103225; CD3-biotin labeled, BioLegend, catalog number 100304; CD3-PE, BioLegend, catalog number 100307; CD3-AF488, BioLegend, catalog number 100321; CD3-AF647, BioLegend, catalog number 100322; CD8-biotin labeled, BioLegend, catalog number 100704; CD8-PECy7, BioLegend, catalog number 100722; CD11b-biotin labeled, BioLegend, catalog number 101204; CD11b-PECy7, BioLegend, catalog number 101216; CD11b-AF488, BioLegend, catalog number 101217; CD11b-AF647, BioLegend, catalog number 101218; CD11c-FITC, BioLegend, catalog number 117305; CD11c-PE, BioLegend, catalog number 117307; CD16/32-PE, BioLegend, catalog number 101308; CD16/32-APCCy7, BioLegend, catalog number 101328; CD24-PE, BioLegend, catalog number 138503; CD24-BV421, BioLegend, catalog number 101825;

	CD31-PE, BioLegend, catalog number 102507; CD31-AF647, BioLegend, catalog number 102516; CD34-FITC, Thermo Fisher Scientific, catalog number 11-0341-85; CD34-efluor 660, Thermo Fisher Scientific, catalog number 50-0341-82; CD41-FITC, BioLegend, catalog number 133904; CD41-BV605, BioLegend, catalog number 133921; CD41-biotin labeled, BioLegend, catalog number 133930; CD42d-F488, Invitrogen, catalog number 53-0421-82; CD42d-APC, BioLegend, catalog number 148506; CD43-PE, BioLegend, catalog number 143209; CD45-PE, BioLegend, catalog number 103106; CD45.1-PE, BioLegend, catalog number 110708; CD45.1-APC, BioLegend, catalog number 110714; CD45.1-AF488, BioLegend, catalog number 110718; CD45.2-FITC, BioLegend, catalog number 109806; CD45.2-APC, BioLegend, catalog number 109814; CD45.2-AF700, BioLegend, catalog number 109822; CD48-AF488, BioLegend, catalog number 103414; CD48-AF647, BioLegend, catalog number 103416; CD71-AF647, BD Biosciences, catalog number 563504; CD71-PE, BioLegend, catalog number 113807; Biotin anti-mouse CD71, BioLegend, catalog number 113803; CD105-PECy7, BioLegend, catalog number 120410; CD115-PE, BioLegend, catalog number 135506; CD115-AF488, BioLegend, catalog number 135512; CD115-BV421, BioLegend, catalog number 135513; CD117-AF488, BioLegend, catalog number 105816; CD117-APCCy7, BioLegend, catalog number 105826; CD117-BV421, BioLegend, catalog number 105827; CD117-PECF594, BioLegend, catalog number 105834; CD117-PECy7, Thermo Fisher Scientific, catalog number 25-1171-81; CD117-BV480, BD, catalog number 566074; CD127-PE, BioLegend, catalog number 135009; CD127-Biotin, Biolegend, catalog number 135005; CD127-BV785, BioLegend, catalog number 135037; CD127-AF647, BioLegend, catalog number 135020; CD135-APC, BioLegend, catalog number 135310; CD144-AF647, BioLegend, catalog number 138108; CD150-PE, BioLegend, catalog number 115904; CD150-AF488, BioLegend, catalog number 115916; CD150-BV421, BioLegend, catalog number 115925; ESAM-PE, BioLegend, catalog number 136204; ESAM-FITC, Biolegend, catalog number 136205; IgM-AF488, BioLegend, catalog number406522;IgD-AF700, BioLegend, catalog number 405729; IgD-AF488, BioLegend 405718; IgM-APC, BioLegend, catalog number 406525; MHC II-biotin labeled, BioLegend, catalog number 107603; MHC II-AF488, BioLegend, catalog number 107616; MHC II-AF647, BioLegend, catalog number 107618; Ly6C-biotin labeled, BioLegend, catalog number 128004; Ly6C-AF647, BioLegend, catalog number 128010; Ly6C-AF488, BioLegend, catalog number 128022; Ly6C-PerCP, BioLegend, catalog number 128028; Ly6G-biotin labeled, BioLegend, catalog number 127604; Ly6G-PerCPCy5.5, BioLegend, catalog number 127616; Ly6G-PECy7, BioLegend, catalog number 127617; Ly6G-AF488, BioLegend, catalog number 127626; Sca1-FITC, BioLegend, catalog number 108106; Sca1-PECy7, BioLegend, catalog number 108114; Ter119-biotin labeled, BioLegend, catalog number 116204; Ter119-AF488, BioLegend, catalog number 116215; Ter119-AF647, BioLegend, catalog number 116218; Ter119-AF700, BioLegend, catalog number 116220; Gr1-biotin labeled, Biolegend, catalog number 108403;
Validation	Antibodies were validated by comparing their staining pattern on mouse BM cells by FACS. For imaging experiments each antibody was validated by testing that the frequency of cells stained matched the one obtained in FACS analyses.

Animals and other research organisms

Policy information about studies involving animals; ARRIVE guidelines recommended for reporting animal research, and Sex and Gender in Research

Laboratory animals	C57BL/6J-Ptprcb (CD45.2+) mice; B6.SJL-PtprcaPepcb/BoyJ (CD45.1+) mice, JAX: 002014; B6.Cg-Ndor1Tg(UBC-cre/ERT2)1Ejb/1J, JAX: 007001;B6.129P2-Gt(ROSA)26Sortm1(CAG-Brainbow2.1)Cle (R26R-Confetti). JAX:017492. Both male and female at 8-14 weeks old for young age mice and 80-100 weeks old for old age mice were used in our experiments.
Wild animals	No wild animals were involved in our study.
Reporting on sex	Both male and female mice were used in our experiments.
Field-collected sample	No field collected samples were used in the study
Ethics oversight	All mouse experiments –except live mouse imaging experiments- were approved by the Institutional Animal Care Committee of Cincinnati Children's Hospital Medical Center. Live mouse imaging experiments were performed in compliance with institutional guidelines and approved by the Subcommittee on Research Animal Care (SRAC) at Massachusetts General Hospital. The following mouse strains were used: C57BL/6J-Ptprcb (CD45.2), B6.SJL-PtprcaPepcb/BoyJ (CD45.1), B6.Cg-Ndor1Tg (UBC-cre/ERT2)1Ejb/1J (Ubc-creERT2), C57BL/6-Tg(CAG-EGFP)131Osb/LeySopJ (Actin-GFP) and B6.129P2-Gt(ROSA)26Sortm1(CAG-Brainbow2.1)Cle (R26R-Confetti). R 26R-Confetti mice were crossed with Ubc-creERT2 mice to generate Ubc-creERT2:Confetti mice. All mice were maintained on a C57BL/6J background. Eight to twelve (2-month-old) and eighty to a hundred weeks (20-month-old) male and female mice were used. All mice were bred and aged in our vivarium or purchased from the Jackson Laboratory. Mice were maintained at the vivarium at Cincinnati Children's Hospital Medical Center under a 14-hours light:10-hours darkness schedule, 30–70% humidity, 22.2 ± 1.1 °C, and specific-pathogen-free conditions.

Note that full information on the approval of the study protocol must also be provided in the manuscript.

Flow Cytometry

Plots

Confirm that:

☒ The axis labels state the marker and fluorochrome used (e.g. CD4-FITC).

☒ The axis scales are clearly visible. Include numbers along axes only for bottom left plot of group (a 'group' is an analysis of identical markers).

☒ All plots are contour plots with outliers or pseudocolor plots.

☒ A numerical value for number of cells or percentage (with statistics) is provided.

Methodology

Sample preparation	Mice were euthanized by isoflurane inhalation followed by cervical dislocation. Bone marrow cells were harvested by flushing bones with 1 ml of ice-cold PEB buffer (2 mM EDTA and 0.5% bovine serum albumin in PBS). Blood was collected from the retro-orbital venous sinus in tubes containing EDTA. Red blood cells in peripheral blood were lysed by the addition of 1 ml of RBC lysis buffer (150 mM NH4Cl, 10 mM NaCO3 and 0.1 mM EDTA). Cells were immediately decanted by centrifugation, resuspended in ice-cold PEB. Cells were stained under dark for 30 min in PEB buffer containing antibodies, washed thrice

with ice cold PBS.

Instrument

Cells were stained in the dark for 30 minutes in ice-cold PEB buffer containing antibodies, washed thrice with ice-cold PEB, and analyzed in an LSRFortessa™ Flow Cytometer (BD Biosciences), LSR II Flow Cytometer (BD Biosciences), or FACS-purified in a FACSAria™ II Cell Sorter (BD Biosciences) or an SH800S Cell Sorter (Sony Biotechnology).

Software

FACSDiva software (BD Biosciences) for data collection and FlowJo (Tree Star) for data analysis.

Cell population abundance

Freshly sorted cells were examined by the same FACS sorter again and the purity is >95%.

Gating strategy

In all experiments, debris were excluded by using Forward scatter/Side scatter (FSC/SSC). Doublets were excluded by double forward (FSC-A and FSC-W), and side scatter (SSC-A and SSC-H). Dead cells were excluded as DAPI+ cells. BM LT-HSC, ST-HSC, MPP2, MPP3, MPP4, MkP, Pre Meg-E, Pre CFU-E, CFU-E, Pre GM, GMP, GP, MoP, MDP, CMP were gated as previously described[1, 2, 3]. Briefly, BM LT-HSC are Lineage-CD117+Sca1+CD135-CD150+CD48-,BM ST-HSC are Lineage-CD117+Sca1+CD135-CD150-CD48-, BM MPP2 are Lineage-CD117+Sca1+CD135-CD150+CD48+, BM MPP3 are Lineage-CD117+Sca1+CD135-CD150-CD48+,BM MPP4 are Lineage-CD117+Sca1+CD135+. Both in the BM and peripheral blood, B cells and T cells were gated as B220+ or CD3+, respectively. Neutrophils were gated as Ly6G+ cells in the peripheral blood. Gating strategies for other cells were detaily described in the manuscript and figures.

1, Yanez, A. et al. Granulocyte-Monocyte Progenitors and Monocyte-Dendritic Cell Progenitors Independently Produce Functionally Distinct Monocytes. Immunity 47, 890-902 e894, doi:10.1016/j.immuni.2017.10.021 (2017).
2, Pronk, C.J., et al., Elucidation of the phenotypic, functional, and molecular topography of a myeloerythroid progenitor cell hierarchy. Cell Stem Cell, 2007. 1(4): p. 428-42.
3, Pietras, E. M. et al. Functionally Distinct Subsets of Lineage-Biased Multipotent Progenitors Control Blood Production in Normal and Regenerative Conditions. Cell Stem Cell 17, 35-46, doi:10.1016/j.stem.2015.05.003 (2015).

☒ Tick this box to confirm that a figure exemplifying the gating strategy is provided in the Supplementary Information.
